# In Sickness and in Health: The Oxygen Reactive Species and the Bone

**DOI:** 10.3389/fbioe.2021.745911

**Published:** 2021-11-23

**Authors:** Joana Reis, António Ramos

**Affiliations:** ^1^ Agronomic and Veterinary Sciences, School of Agriculture, Polytechnic Institute of Viana Do Castelo, Ponte de Lima, Portugal; ^2^ TEMA, Mechanical Engineering Department, University of Aveiro, Aveiro, Portugal

**Keywords:** oxidative stress, bone, mechanobiology, redox balance, mechanotransduction

## Abstract

Oxidative stress plays a central role in physiological and pathological bone conditions. Its role in signalment and control of bone cell population differentiation, activity, and fate is increasingly recognized. The possibilities of its use and manipulation with therapeutic goals are virtually unending. However, how redox balance interplays with the response to mechanical stimuli is yet to be fully understood. The present work summarizes current knowledge on these aspects, in an integrative and broad introductory perspective.

## Introduction

Oxidative stress plays an important role in homeostasis and disease in most tissues. Reactive species are continuously generated as byproducts of normal cellular metabolism. Antioxidant mechanisms, acting *via* specific scavenger reactions and detoxification pathways, revert its accumulation and avoid oxidative stress-related damage (Reis et al., 2021). Curiously, for some processes, the existence of a transitory oxidative stress is a biological necessity. However, when oxidative stress remains because of an imbalance between the production and scavenging of reactive species, it results in an array of physiopathologic changes, consequence of a spiral of new sources of free radicals and oxidative species and increased damage (Reis et al., 2021).

In the literature, the term reactive oxygen species (ROS) is often used to encompass all reactive species, regardless of the specific chemical species. Other authors differentiate reactive nitric species (RNS) when referring to nitric oxide and nitrogen dioxide free radicals, peroxynitrite, and nitrite/nitrate (Sies et al., 2017). ROS are mainly produced by mitochondria, the foundational organelle for energy generation in cells, intervening in many of the cell signaling cascades (McBride et al., 2006; Quirós et al., 2016; Zheng et al., 2020). The most important oxygen free radicals include hydrogen peroxide (H_2_O_2_), hydroxyl radical (-OH), superoxide anion radical (·O_2_ −) and nitric oxide (NO). However, other oxygen-derived free radicals have relevant roles in cell metabolism, such as the peroxyl radical cation and other hydroperoxides (Dröge, 2002).

Oxidative stress is being increasingly recognized by its dual role, no longer the cause and root of all evil. ROS generation is not only essential as part of the immune cells’ response against pathogens but may act to signal and modulate cell responses, essential for life. Oxidation–reduction (redox) homeostasis is ubiquitous to living cells, tissues, organs, systems. Oxidative eustress or physiological oxidative stress is positive and a fundamental signal and control mechanism (Sies et al., 2017); low concentrations of ROS and RNS allow reversible oxidative/nitrosative modifications of redox-sensitive residues in regulatory proteins; these modifications may translate into a loss or gain of function or a change of function (Dröge, 2002; Moldogazieva et al., 2018). Oxidative distress is associated with high burden, supraphysiological oxidative challenge and has deleterious consequences, leading to oxidative damage of biomolecules and disruption of the redox signaling pathways (Sies, 2019).

The skeletal system shares with the cardiovascular, muscular, and connective tissues its embryonic mesodermal origin; thus it is possible these tissues share some regulatory pathways. NO has an essential function in cardiovascular function. The nitric oxide synthase (NOS) in the endothelial cells regulates vascular smooth muscle relaxation through nitric oxide synthesis, mediates angiogenesis, and controls muscle cell proliferation; diffused NO inhibits platelet aggregation and thrombogenesis (Farah et al., 2018). In blood vessels, NO is released in response to stimuli such as shear stress. It has been hypothesized that the signaling pathways leading to anti-atherogenic or pro-atherogenic vascular wall reactions are ROS/RNS dependent and depend on the flow patterns (Hsieh et al., 2014); the endothelial cells are sensitive to fluid shear and several processes, including vascular tone and remodeling, angiogenesis and vascular morphogenesis are modeled by these forces and the resulting cell response (Roux et al., 2020).

Bone is highly dynamic, its form and cellular activity continuously tailored by load and strain, responsive to external and internal stimuli. Environment, cell-to-cell, and cell-matrix interactions regulate osteogenesis, bone repair, and remodeling, a process coupling bone resorption and formation. Bone homeostasis is strongly intertwined with intracellular reactive species production, namely reactive oxygen species (ROS) and reactive nitric species (RNS).

This paper intends to condense the current knowledge on the role of the redox balance in physiological and pathological conditions in bone, as well in the presence of orthopedic implants, and establishing connections to bone mechanobiology, still largely unexplored in this aspect. It is aimed at those that, like the authors, come from a clinical or mechanical and biomedical engineering background.

## Oxidative Stress in Bone Homeostasis

**FIGURE 1:**
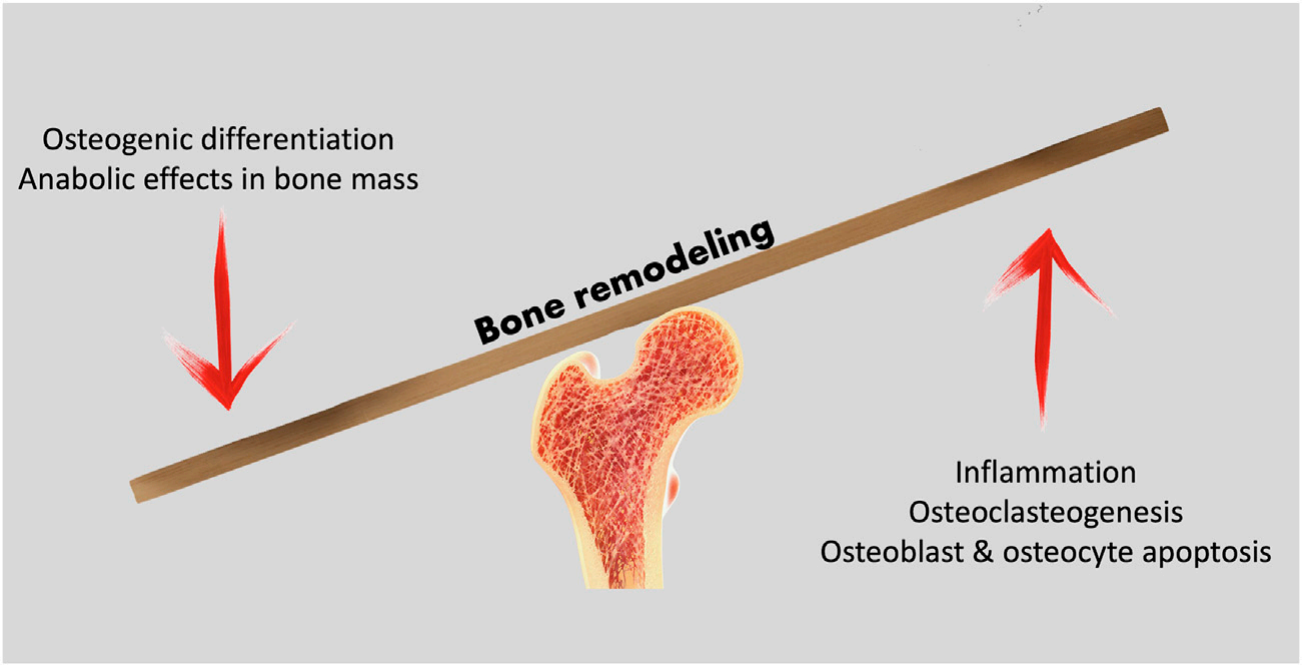
Bone remodeling processes rely on the balance between anabolic and catabolic cell activity to maintain a healthy bone structure, adapted to loading demands.

Mature bone contains three key cell populations: osteoblasts, osteocytes and osteoclasts. While osteoblasts differentiate from mesenchymal stem cells, and may differentiate into osteocytes, osteoclasts arise from the same lineage as macrophages and monocytes. The bone remodeling process depends on the coordinate action of the different cell populations; osteoblastic bone formation activity must be balanced by osteoclastic resorptive action (Figure 1). Osteoblasts and osteocytes express membrane-bond RANKL (receptor activator of nuclear factor NF-κB ligand) and this regulatory molecule interacts with a receptor - RANK (receptor activator of nuclear factor-κB) -, expressed on the surface of osteoclast precursors. RANK activation by RANKL is essential for fusion of the osteoclast precursor cells and osteoclast formation (da Costa Reis & Oliveira, 2020; Nakashima et al., 2011). Osteoblasts also secrete osteoprotegerin (OPG), an inhibitor of osteoclastogenesis. The Wnt/β-Catenin pathway is fundamental for bone-mass homeostasis; osteocytes are key to the canonical Wnt signaling pathway regulation, for they produce Wnt ligands, are targeted by these and secrete molecules that modulate Wnt actions (Al-Bari & Al Mamun, 2020).

NO and prostaglandin E2 (PG E2) are released by osteoblastic lineage cells under cyclic mechanical load; strain applied through the substrate and through fluid flow stimulated the release of nitric oxide (Mullender et al., 2004; Frias et al., 2010; Yavropoulou and Yovos, 2016). Nitric oxide and PG E2 are essential for balanced bone remodeling since both are related to RANKL expression (Huang et al., 2017; I; ntemann et al., 2020). PG2 induces RANKL expression in osteoblasts, in an autocrine and paracrine manner *via* activation of the EP4 receptor, and exerts regulatory action on angiogenesis and vascular permeability, thus, modulating bone metabolism (Intemann et al., 2020).

Superoxide synthesis by the osteoclasts’ NADPH oxidases (Nox) is also necessary to allow bone resorption in physiological bone remodeling processes (Darden et al., 1996). There are three identified Nox isoforms involved in osteoclast differentiation and function, Nox1, Nox2, and Nox4. Nox isoforms expression is controlled by several mechanisms such as the transcription factor nuclear factor-erythroid 2-related factor (Nrf2), key to antioxidant cellular responses, and regulator of bone homeostasis (Sun et al., 2015; Wegner and Haudenschild, 2020). Nuclear respiratory factor 1 (Nrf1) is also a transcription factor, of the same family, and known to regulate the antioxidant response elements-driven target genes (Xing et al., 2007). Both have been linked to the expression and activity of Osterix and RUNX, both associated with osteoblast differentiation and bone metabolism regulation (Xing et al., 2007; Sun et al., 2015).

**FIGURE 2:**
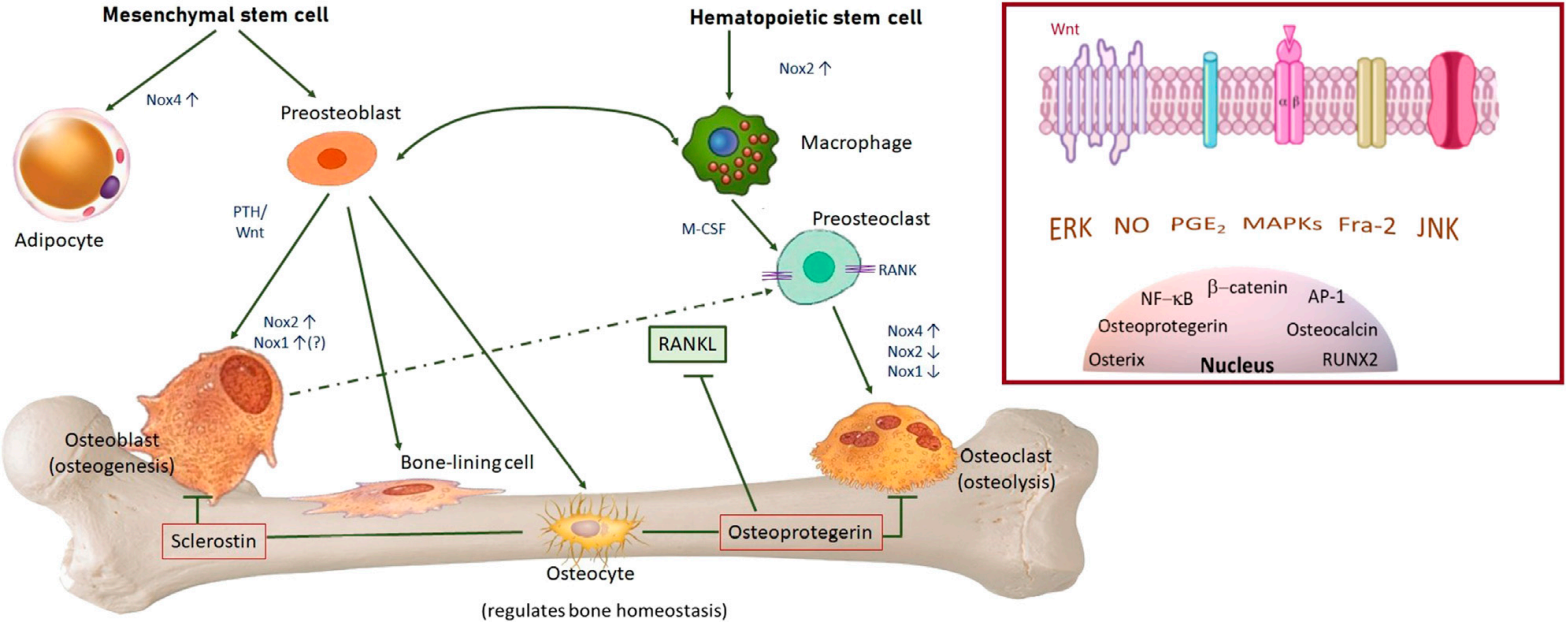
Main cellular populations in bone, role and NADPH oxidases isoforms expressed, paramount for ROS-mediated differentiation, activity and fate of osteoblasts and osteoclasts. Under mechanical loading, Nox2 is activated in osteocytes, producing ROS and leading to ROS-mediated decrease in sclerostin and activation of the Wnt/β-catenin pathway. Bone marrow macrophages express Nox2. During RANKL-induced osteoclast differentiation, Nox2 is suppressed and Nox4 is upregulated by RANKL. Overexpression of Nox4 increases adipogenesis and not osteogenesis. Osteogenic differentiation involves parathormone (PTH) and Wnt signalling pathways. Nox1 and Nox2 are inducible and thought to have a role in osteoblast proliferation and differentiation. Osteoprotegerin expression is induced by physiological mechanical stimulation and inhibits osteoclasteogenesis. Nox4 synthetizes H_2_O_2_ and drives cell differentiation. Insert: bone cell regulation and mechanotransduction involves several ROS-sensitive pathways.

While ROS generation in inflammatory processes arises mainly from the mitochondria, fine-tuned ROS generation, both in physiologic and pathologic conditions, is originated mainly by members of the Nox family; these differ in cellular location, activation mode and type of ROS they produce; Nox1/2 produce •O_2_ˉ and Nox4 produces H_2_O_2_, contributing to modulate the formation, activity, and survival of osteoblasts and osteoclasts (Figure 2).

Osteocyte differentiation has been associated with increased Nrf2 activity, that responding to raised ROS levels, drives the transcription of osteocyte-specific genes. This is likely caused by increased mitochondrial numbers associated to glucose deprivation. Nutrient deprivation likely results from the osteoblast entrapment in the mineralized bone matrix, triggering the osteoblast-osteocyte transition (Sánchez-de-Diego et al., 2021). Thus, cell fate (osteocytogenesis or apoptosis) is again finely tuned by the cell redox state.

**FIGURE 3:**
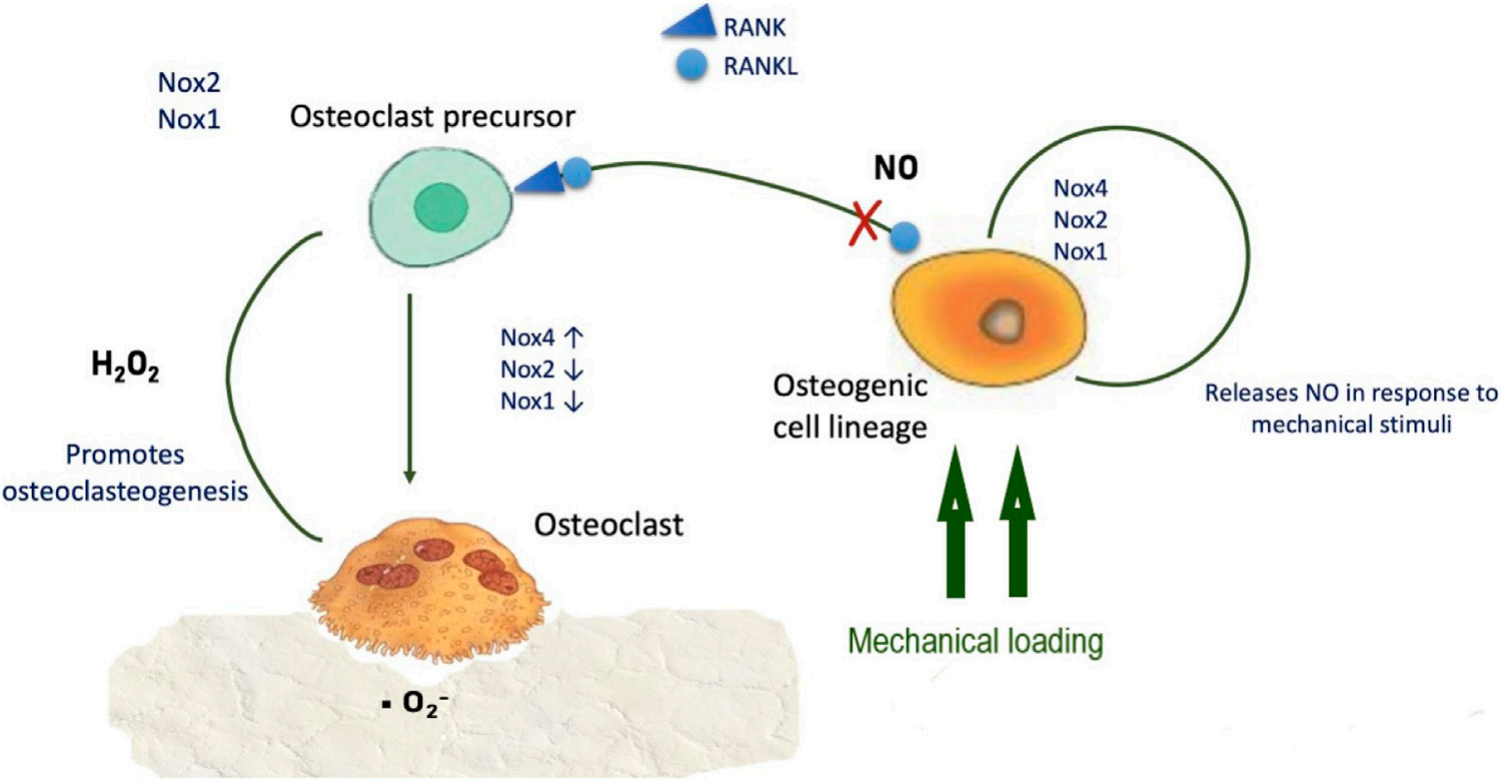
ROS synthesis and regulation of osteogenic and osteoclastic cells. Physiological levels of NO inhibit RANK/RANKL-mediated osteoclastogenesis.

NO is essential to control and balance periodontal stem cell differentiation, promoting osteogenic differentiation rather than adipogenic (Yang et al., 2018), and has anabolic effects in bone, promoting osteoblast differentiation and glucose metabolism. NO production depends on arginine synthesis *via* the enzyme argininosuccinate lyase and its production is negatively modulated by control mechanisms such as nitric oxide synthase binding by caveolin-1 (Jin et al., 2021). NO release may be triggered by RANKL; since inhibition of RANKL-induced NO increases osteoclastogenesis and bone resorption, osteoclastogenesis in response to RANKL is probably diminished by NO production (Huang et al., 2017) (Figure 3).

The transcription factor activator protein 1 (AP-1) is implicated in differentiation mechanisms and cell activity regulation (Garces de Los Fayos Alonso et al., 2018; Bejjani et al., 2019). It is formed by c-Fos and c-Jun proteins and its activity is redox-regulated (Abate et al., 1990). Intermediate levels of ROS activate the AP-1 transcription factor and the NF-κB signaling pathway, while low levels of ROS stimulate Nrf2. NF-κB activation triggers osteoclastogenesis and increased levels of inflammatory cytokines (Le Rossignol et al., 2018; Lepetsos et al., 2019). NO may also suppress the DNA-binding activity of AP-1 through S-glutathionylation; NO modifies the two cysteine residues contained in the DNA binding module of c-Jun (Klatt et al., 1999; Lepetsos et al., 2019), supporting NO anabolic effects on bone mass. High AP-1 is, thus, one of the transcription factors intervening in osteoclastogenesis and in the regulation of osteoclast activity (Wagner, 2010; Pang et al., 2019). c-Jun and AP-1 are also involved in osteogenic differentiation of mesenchymal stem cells, namely through increased RUNX2 expression (Fu et al., 2019). Under or over-expression of Fra-2, a Fos-related protein of the AP-1 family, results in structural and functional bone anomalies. Fra-2 promotes osteoblast differentiation, collagen, and osteocalcin production, apparently in detriment of adipocyte formation, without affecting osteoclastogenesis (Bozec et al., 2010). Recent studies suggest reduced ROS production inhibits osteoclast differentiation and bone resorption (Ohyama et al., 2018), *via* a decrease in mitogen-activated protein kinases (MAPKs) extracellular signal-regulated Erk and the activation of c-Jun N-terminal kinases (Erk). The elevation of intracellular ROS levels leads to the activation of MAPKs, such as p38, Erk, and JNK, by oxidative modifications of MAPK signaling proteins and through inactivation of the MAPK phosphatases (MKPs). The latter inactivate MAPKs by dephosphorylation. MKPs may be inactivated by oxidation of cystein residues in the enzyme catabolic site. However, MKPs induction or inactivation by ROS is likely dependent on ROS levels. ROS may simultaneously activate MAPKs and induce MPK expression (Son et al., 2013).

H_2_O_2_ promotes osteoclast formation and osteoblast apoptosis, inhibiting osteoblast proliferation, while •O_2_ˉ increases with osteoclastic resorption activity, prompted by parathormone and interleukine 1 (Garrett et al., 1990; Lean et al., 2005; Dai et al., 2017).

Oxidative stress impairment of osteoblast differentiation and osteogenic capacity has been associated with bone loss in aging. In the aging mice, the expression of Forkhead box O (FoxO) target genes increases, while the expression of Wnt target genes decreases, due to interference with the Wnt/β-Catenin pathway (Almeida et al., 2007). The FoxO transcription factors regulate the expression of genes coding for proteins with antioxidant activity. FoxO activity may be regulated by posttranslational modifications, protein-protein interactions, and also by mechanisms regulating FoxO gene transcription and mRNA stability. FoxO transcription factors are subject to redox regulation through phosphorylation (through MAPKs activity) and acetylation and ubiquitination of the lysine residues in FoxO proteins (Klotz et al., 2015). β-catenin is required for FoxO transcription, and this binding is stimulated by ROS, thus decreasing β-catenin availability, to the detriment of the Wnt/β-catenin signaling pathway. In aging mice, NAD+ is decreased. NAD + -dependent Sirtuin1 deacetylates FoxOs and β-catenin, increasing Wnt pathway expression in osteoblast progenitors (Kim and Park., 2021). ROS interference in osteoblastogenesis and osteoclastogenesis is also present in pathological conditions such as osteoporosis and arthritis (Agidigbi and Kim, 2019; Wang Y. N. et al., 2021).

The different bone cell types communicate through Cx43 gap junction channels and hemichannels; the Cx43 gap junction channels are central to mechanotransduction and bone remodeling. Under oxidative stress or diminished antioxidative defense conditions, such as osteoporosis due to aging, estrogen deficiency or glucocorticoid treatment, Cx43 expression is decreased. The opening of Cx43 hemichannels has a protective role against osteocyte cell damage by ROS (Hua et al., 2021) and protects the trabecular bone against catabolic effects associated with estrogen deficiency (Ma et al., 2019).

## Bone Development and Healing: The Role of Oxidative Stress

**FIGURE 4:**
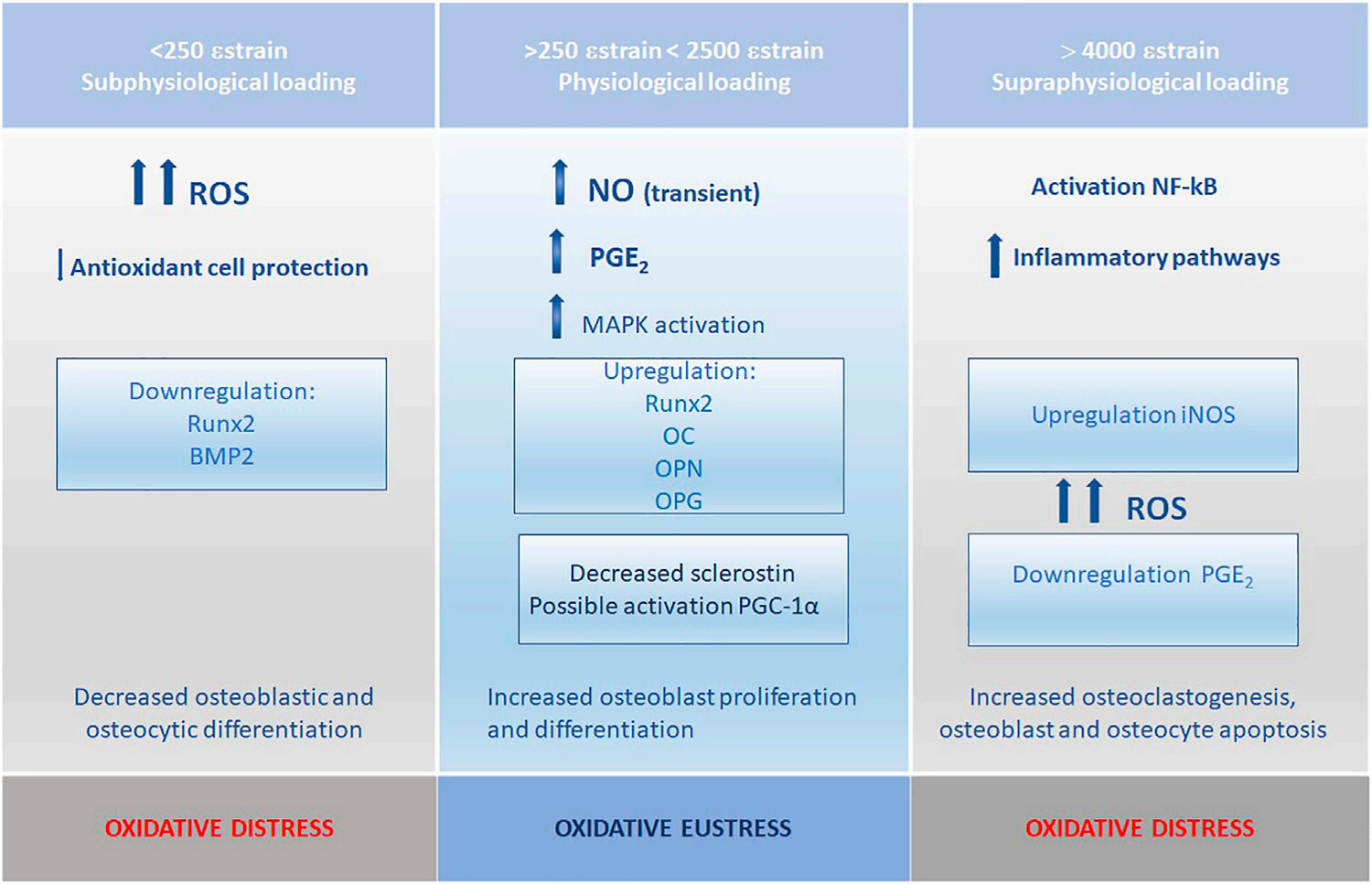
Redox state and strain levels; persistent sub or supraphysiological mechanical stimulation leads to increased ROS levels and deregulation of the bone remodeling and formation processes.

There is evidence that redox states within the embryo shape gene expression patterns through redox-sensitive transcription factors (Figure 4), as has been theorized; changes in redox state may also contribute to spatial differences in cell activity, contributing to cell differentiation, and intimately related to the production of necessary energy for survival and growth (Harvey et al., 2002). Life emerged and evolved in an environment with varying levels of oxygen, often hypoxic, and redox/hypoxia seems to be an ancient and well-conserved regulatory pathway, fundamental for development and regeneration (Loenarz et al., 2011; Coffman and Su, 2019). Mammalian embryos develop in a hypoxic environment; hypoxia inducible factor (HIF) has several isoforms from which HIF1 is the most relevant in the context of skeletal tissues. HIF consists of two subunits, one unstable (HIF-α), and one stable (HIF-β); it is a transcription factor intervening in the cell response to hypoxia. Oxygen concentrations regulate the level and activity of HIF-α through hydroxylation of prolyl residues, by members of the prolyl hydroxylase domain (PHD) family and asparaginyl hydroxylase (FIH) (Kaelin and Ratcliffe, 2008). Under normal oxygen conditions, the hydroxylation of HIF-α decreases its transcriptional activity. Under hypoxia, the hydroxylation of HIF-α is inhibited, since it needs oxygen and iron. HIF-α becomes abundant in the cytoplasm, translocates to the nucleus, and dimerizes with HIF-β subunit. The dimer then binds to hypoxia-response elements (HREs), promoting the expression of target genes that modulate cell response to hypoxia (Coffman and Su, 2019). The formation of vertebrates’ endochondral bone is initiated by the formation of avascular mesenchymal condensations, followed by chondrogenesis, a process in which HIF-1α is required for chondrocyte differentiation, survival, and proliferation (Cordeiro and Tanaka, 2020). HIF has been linked to upregulation of angiogenesis and secretion of extracellular matrix proteins, including collagen type II (Wan et al., 2010; Bentovim et al., 2012). Interestingly, HIF-1α is activated by NO under normoxic conditions (Wan et al., 2010).

Mesenchymal stem cell proliferation and osteogenic differentiation also depend on other transcriptional factors such as Runx2, Osterix, and FoxO, as previously discussed. The above-mentioned transcriptional factors, all directly or indirectly regulated by oxygen and redox environment, do not operate in tightly separated pathways, contributing to the redundancy and multi-point pathway control of cell fate and stress response.

Gene expression is also directly controlled by oxygen levels since histone demethylases are oxygen-dependent, in an oxygen-mediated mechanism that seems to precede the HIF pathway (Coffman and Su, 2019).

Adequate nutrient and oxygen support is, therefore, fundamental for cell fate determination, as it is widely recognized in clinical practice. Preserving vascularization and blood supply to fracture sites is a priority during the surgical approach and osteosynthesis. The mechanical stability of the fracture site is determinant for the healing mode. In stabilized, non-complicated fractures, mesenchymal stem cells differentiate directly into osteoblasts, and fracture healing ensues by intramembranous ossification while if the fracture is unstable, the bone heals through endochondral ossification (Miclau et al., 2017). In most clinical situations, both ossification modes occur. After a bone fracture, there is always some degree of disruption of the blood supply, leading to local lower oxygen levels. This hypoxic local environment is a powerful trigger for neovascularization through the HIF-1 pathway and the expression of vascular endothelial growth factor (VEGF) (Shen et al., 2009). Hypoxia also induces bone morphogenetic protein 2 (BMP2) expression by mesenchymal stem cells through a pathway independent from HIF-1; BMP2 is a powerful inductor of osteo and chondrogenesis, and its induction happens through a redox-sensitive mechanism; if hypoxia is prevented, BMP2 secretion is inhibited and healing is impaired, as happens in fracture non-union (Muinos-López et al., 2016). BMP2 secretion and HIF1 activation seem to overlap. The hypoxia, although necessary, should be transient; normoxia conditions are necessary for collagen cross-linking and stabilization of the basement membrane of the new vascular network that will ensure adequate perfusion of the site (Miclau et al., 2017). NO has a recognized role in fracture healing; NO mediates vasodilation, essential for increased blood flow to the fracture site but also for the vascular response, bone formation, and resorption during the remodelling phase (Ding et al., 2018).

## Oxidative Stress Mechanisms in Disease: How Is Bone Affected

ROS are increased in inflammatory conditions and in a number of non-inflammatory systemic diseases that impact bone metabolism. However, ROS-sensitive mechanisms are also essential for normal fracture healing. Research has been focused on the effects of the superoxide anion and hydrogen peroxide on bone cell function and bone remodeling (Wauquier et al., 2009), but other ROS, as well as the antioxidant systems, are also relevant in disease processes.

Fracture non-union is a frequent, painful, complication of bone fractures, deleterious to patient wellbeing. The work developed by Muinos-López et al. (Muinos-López et al., 2016) suggests that although hypoxia is fundamental for initiation of the fracture healing process, factors scavenging ROS are critical for in early phases and mesenchymal stem cells redox state is determinant. Decreased BMP-2a impairs MSC differentiation and delays cartilage mineralization while elevated metalloproteinases contribute to BMP degradation, as reviewed by Ding et al. (Ding et al., 2018). When considering ROS, although the beneficial role of NO is recognized, it is also known that NO levels are increased in hypertrophic non-union calluses, suggesting NO is part of the molecular pathogenesis of nonunion, a hypothesis also supported by the altered levels of the amino acids associated with NO metabolism in atrophic non-union calluses, with arginine availability seemingly a limiting factor (Wijnands et al., 2012). NOS-knockout mice show impaired fracture healing, through deregulation of arginine-NO metabolism, paired with increased neutrophil influx to the fracture site; this study focused on the activity of NOS2 and NOS3, not of NOS1, the most relevant for the latter remodeling phase (Meesters et al., 2016).

Osteoarthritis (OA) is the most common joint disorder and affects both articular cartilage and subchondral bone. Its etiology is multifactorial but includes ROS overproduction, associated with Nox4 (Drevet et al., 2018), affection intracellular signaling, impairs chondrocyte and matrix metabolism, contributes to inflammation and subchondral bone lesions. The mechanisms by which ROS are involved in the pathogenesis of the articular cartilage lesions are better characterized than those concerning the subchondral bone. In OA, a central role is played by the NF-κB transcription factors family. The NF-κB dimers, when able to translocate from the cytoplasm into the nucleus, regulate the expression of proinflammatory cytokines, immunomodulatory proteins, and molecules vital for cell adhesion and proliferation. NF-κB is redox-sensitive and its activity may be increased or inhibited by ROS, following oxidation or S-glutathionylation of redox-sensitive cysteine residues, depending on the level of ROS, the types of stimuli, and the cell type (Lepetsos et al., 2019). NF-κB may also regulate Nrf2 transcription and activity, affecting the redox balance (Choi et al., 2019; Lepetsos et al., 2019). Chondrocytes treated with advanced oxidation protein products increase the expression of interleukin (IL)-1β and tumor necrosis factor (TNF)-α, known to prompt articular degenerative changes; this happens *via* the Nox4-dependent and p38-MAPK mediated pathway (Liao et al., 2020). Mechanical stress has been associated with the development of OA, *via* activation of interleukin-1β, tumour necrosis factor-α, nuclear factor kappa-B, Wnt, transforming growth factor-β, microRNAs pathways, and the oxidative stress pathway. Involved receptors include integrin, ion channel receptors, hydrogen peroxide-inducible clone-5, Gremlin-1, and transient receptor potential channel 4 (Fang et al., 2021).

Rheumatoid arthritis (RA) is a chronic systemic autoimmune disease, arising from the synovia; it may course with synovial hyperplasia, cartilage damage, bone erosion, and systemic repercussions. The subchondral bone may become eroded as a result of increased numbers of osteoclasts and decreased osteoblasts (Guo et al., 2018). RA is associated with high levels of ROS and local bone loss as a consequence of inflammation. Redox-sensitive transcription factors, including NF-κB, AP-1, and Nrf2, are involved in the pathogenesis of RA (Le Rossignol et al., 2018). NOX4 is the only NOX isoform found in human chondrocytes and it may be central in cartilage degradation and development of osteoarthritis (Agidigbi and Kim, 2019).

Osteoporosis, another multifactorial progressive disorder, is not inflammatory in nature, as opposed to OA and RA. However, there is increasing evidence that redox imbalance is implicated. In patients with post-menopausal osteoporosis, plasma total oxidative status and oxidative stress index were significantly higher than in healthy controls, and total antioxidant status was lower (Altindag et al., 2008). NOX4 is involved in bone loss and represents a potential therapeutic target for the treatment of osteoporosis. Inhibition of Nox4 activity attenuates osteoclastogenesis, which is accompanied by impaired activation of RANKL-induced NFAT-1 (nuclear factor of activated T cells 1) and c-Jun (Schröder, 2015). NFATc1 signaling could be the key downstream event in RANKL-mediated ROS signaling (Agidigbi and Kim, 2019). Age-related osteoporosis is also related to a deficit in osteoblasts, which decline in numbers with aging. In aged mice, decreased levels of NAD+ were described, associated with increased acetylation of the FoxO1/β-catenin pathway and markers of cell senescence. Reduction of NAD + levels in osteoprogenitor cultures from young mice inhibited osteoblastogenesis in a FoxO-dependent manner (Kim et al., 2021).

Diabetes mellitus (DM) is an important cause of impaired bone healing and delayed fracture healing; recent studies suggest high glucose concentrations are deleterious for osteoclastogenesis and osteoclast function (Hu et al., 2019). High glucose levels also suppress osteogenic differentiation *in vitro* by promoting the production of ROS and downregulating the anti-oxidative defense enzyme superoxide dismutase; these effects are reversed by the use of antioxidants (Dong et al., 2017). Low bone mineral density has been reported associated with DM (Adil et al., 2015) and differences may be more marked in post-menopausal women with DM (Halper-Stromberg et al., 2020). These anomalies have been associated with increased ROS by altered energy metabolic pathways (Bacevic et al., 2017; Dong et al., 2017; Korac et al., 2021).

The skeleton is affected by primary and secondary neoplasia. As in healthy tissue, ROS are important determinants of cancer biology and behavior. *Cancer* cells produce ROS and these may act promoting cell survival and proliferation or cell death. In osteosarcoma, the knockdown of NOX2 significantly suppressed ROS generation, inducing apoptosis as a result (Kitamoto et al., 2018). Nrf2 is related to a poor prognosis in osteosarcoma (Park et al., 2012); Nrf2 heightened activity protects cells against apoptosis, even following DNA damage which is not adequately corrected; tumorigenesis may also occur due to metabolic changes initiated by Nrf2 activation or by signaling changes that are Nrf2-dependent (Gunne et al., 2020). Nfr2 promotes cancer development after it is established, a change to its protective role in the physiological status. Nfr2 also modulates the immune response; immune suppression in cancer depends mainly on regulatory T (Treg) cells and myeloid-derived suppressor cells (MDSCs) (He et al., 2020). The inhibition of the cytoprotective, antioxidant Nfcr2 pathways has been shown to be a highly promising therapeutic solution for numerous tumors, including osteosarcoma (Lu et al., 2018; Panieri and Saso, 2019; Telkoparan-Akillilar et al., 2021). Solute carrier 25 family member 10 (SLC25A10), or dicarboxylate carrier, also is important for redox homeostasis. SLC25A10 levels are elevated in human osteosarcoma tissues, compared with normal bone tissues; higher SLC25A10 levels have also been positively correlated with metastization (Wang et al., 2020).

## Oxidative Stress and Bone Mechanobiology

Adequate mechanical stimulation is of paramount importance for bone homeostasis, remodeling, and formation. As postulated by Wolff’s law, bone adapts to functional loads conditions, in such a way that its mass and architecture are optimal; the magnitude and distribution of resulting strains are decisive (Rubin and Lanyon, 1984). Bone adaptation occurs to maintain the local strain; increased bone strain from physical activity may induce bone gain, if within the elastic deformation interval, while decreased bone strain results in bone loss (Sugiyama et al., 2016). However, mechanical loading influences more than bone mass and microarchitecture, since loading also increases bone material strength in post-menopausal healthy women; the effects of the daily one-leg jump, in increasing number for 3 months, was present on the loaded tibial bone when compared to contralateral control tibia (Sundh et al., 2018).

Supraphysiological mechanical forces induce inflammation through activation of the NF-κB cascade, known to be redox-sensitive (Chatterjee and Fisher, 2014). Excessive mechanical loading induces an increase in ROS, activating NF-κB and cartilage degeneration (Chang et al., 2019), further supporting the existence of a ROS-mediated mechanism in damage associated with mechanical stress. Lack of sufficient mechanical stimuli induces altered mitochondrial function. Microgravity conditions inhibit the proliferation of mesenchymal stem cells and osteogenic differentiation through downregulation of a multitude of genes, including Runx2 and BMP2 (Li et al., 2019). Microgravity deeply affects osteoblast mitochondrial energy potential, inducing an oxidative stress response, with decreased oxidized glutathione and antioxidant enzymes (Michaletti et al., 2017); similar interference with the glycolysis pathways, resulting in downregulation of osteocytic genes, was described in osteocytic cell lines under microgravity conditions (Uda et al., 2021). Mechanical unloading increased both intracellular ROS production and the Sod1 expression in bone tissue including bone marrow cells in mice (Morikawa et al., 2013).

However, again ROS effects are dual. Both NO and PG E2 are required in mechanically induced bone formation (Chow et al., 1998; Watanuki et al., 2002) (Figure 4). The nitric oxide synthase has several isoforms: an endothelial isoform (eNOS), extensively expressed in bone on a constitutive basis, and an inducible isoform (iNOS), only expressed in response to inflammatory stimuli and a neuronal NOS (nNOS). The eNOS isoform plays a key role in regulating osteoblast activity and bone formation also mediating bone mechanical stimulation (van’t Hof and Ralston, 2001; Schröder, 2015). Osteocytes express NO transporters and a few minutes after a mechanical stimulus, release NO which suppresses bone resorption (Chambers et al., 1999). Increased ROS levels and osteogenic gene expression in MC3T3-E1 osteoblasts, through MAPK activation mediated by ROS, were described after stimulation by low-intensity pulsed ultrasounds; inhibition of ROS attenuated osteogenic gene expression, including Runx2, osteocalcin, and osteopontin (Kaur et al., 2017). It is also recognized that osteocytes transduce mechanical load signals to activate Nox2, producing ROS signals; this response is accompanied by a rapid ROS-mediated decrease in sclerostin levels by lysosomes, allowing activation of the Wnt/β-catenin signaling pathway, and bone formation (Schröder, 2015; Gould et al., 2021). However, in endothelial cells, oxidative stress due to unregulated Nox activity leads to altered eNOS function, shifting from NO production to O_2_^−^, further inducing oxidative stress and increasing ROS levels, with subsequent endothelial disfunction (Meza et al., 2019). It is possible that a similar process occurs in bone.

The peroxisome proliferator-activated receptor-gamma, coactivator 1 alpha (PGC-1α) is an important regulator of mitochondrial biogenesis, and it regulates ROS metabolism by preventing oxidative stress. In skeletal muscle, its role in mitochondrial homeostasis during differentiation has been recognized. Downregulation of PGC-1α caused impairment of antioxidants expression, accompanied by a significant burst in ROS and oxidative damage to proteins. Mitochondrial mass and function decreased while mitophagy augmented through the ROS/FOXO1 pathway (Baldelli et al., 2014). The PGC-1α anabolic role in bone has been highlighted by a number of *in vitro* and *in vivo* studies, as recently reviewed by Buccoliero et al., (Buccoliero et al., 2021). Sirtuin 3, a mitochondrial (NAD)-dependent deacetylase, is a key regulator of osteoblastic differentiation through regulation of mitochondrial function; its absence reduces the expression of superoxide dismutase 2 (Sod2), a mitochondrial molecule with antioxidant activity that permutes superoxide into the less reactive hydrogen peroxide. PGC-1α stimulates Sirtuin 3 activity on osteogenic differentiation (Buccoliero et al., 2021). How different biophysical stimuli may affect mitochondrial metabolism bone was recently reviewed by (Wang F.-S. et al., 2021). In rat skeletal muscle tissue, the expression of PGC-1α and HIF-1α were differently affected by the training regimen (continuous, moderate exercise vs high-intensity interval) (Ahmadi et al., 2021). However, regarding bone, there is still limited information on how different loading regimens affect these pathways.

## Oxidative Stress and Orthopedic Implants

Inflammation and hypoxia are leading conditions that drive oxidative stress (Mittal et al., 2014). Neutrophils, eosinophils, and monocytes/macrophages are able to produce large amounts of ROS such as superoxide and hydrogen peroxide as part of the response to a perceived pathogen invasion or mechanical trauma; the release of ROS may be further stimulated by inflammatory mediators (Hameister et al., 2020).

Following trauma, bone healing is initiated with hematoma formation and acute inflammatory response. Disruption of the vascular and bone structures triggers the release of cytokines, leading to inflammatory cell recruitment, especially neutrophils and macrophages (Bahney et al., 2019). Macrophage differentiation and function are strongly influenced by ROS (Zhang et al., 2013). Interestingly, macrophages present different gene expression patterns and cytokine secretion profiles, depending on whether they were induced in pathological conditions or RANKL-induced M1 macrophages. These were found *in vivo* close to iNOS + cells (inducible nitric oxide synthase-positive cells), peaking on day 7 during bone healing, suggesting they are NO-dependent, involved in bone formation. M1 macrophages’ activity is both influenced and influences the microenvironment (Huang et al., 2017).

Surgical trauma is, thus, a cause of oxidative stress, with its magnitude influenced by pathology and comorbidities (Rosenfeldt et al., 2013; Karachalios et al., 2021). Prior to implantation, patients with orthopedic disease often suffer from conditions that contribute to oxidative stress, affect the local microenvironment, and may condition surgical success and implant performance (Mouthuy et al., 2016). The implant itself may drive further oxidative stress, as well as suffer degradation in consequence (Mouthuy et al., 2016; Borys et al., 2018; Eliaz, 2019). Implant wear products are a known cause for inflammation and ROS generation; ROS are generated by local macrophages, through the NOX signaling pathway, also affecting the NF-κB activation (Chen et al., 2015). Although ROS originate from acute and chronic inflammation, they may further arise from the metal surfaces of implants by reduction reactions, subsequently affecting neighboring cells. In turn, the lower pH associated with inflammation is a contributor to corrosion phenomena; the resulting increase in released particles will perpetuate inflammation, further implant structural damage, and eventually promote systemic effects (Eliaz, 2019; Hameister et al., 2020). Ferroptosis is a form of controlled cell death that occurs in the presence of iron overload and leads to the formation of lipid ROS (Sharma and Flora, 2021), and its association with peri-implant inflammation and cell death has been suggested (Liu et al., 2020). Likewise, increased local ROS levels, inflammation and metallosis have been associated with titanium alloy implants and tribocorrosion phenomena (Borys et al., 2019; Eliaz, 2019).

The performance of implants is also greatly influenced by protein adsorption to the implant surface, with an effect on its stability, metal ion release, and cell adhesion; the redox balance influences protein-metal interactions; recent studies show H_2_O_2_ impedes the formation of dense protein domains on Ti6Al4V surface, increasing protein adsorption, surface potential and total roughness in Ti6Al4V implant surface (Rahimi et al., 2021).

## Discussion

Oxidative stress has been one of the factors implicated in bone disease, in the loss of biomaterials biocompatibility and function. It is now clear that redox mechanisms are paramount for bone physiology.

A better understanding of the host-implant interplay and the role of reactive species and oxidative stress regarding the fate of implanted biomaterials is necessary.

Comorbidities such as DM, OA, and RA may also contribute to oxidative stress since in all these diseases, ROS are increased and NF-κB is activated, leading to inflammation and imbalances in the bone coupling processes. Excessive production of ROS in these pathologies results from abnormal oxidative energy metabolism in the mitochondria, the activity of enzymatic complexes like the Nox, or both. Noxs, namely Nox4, are one of the culprits (Wauquier et al., 2009; Korac et al., 2021). DM, OA, and RA may be present prior to the surgical procedure, influencing the local microenvironment and thus, the clinical outcome (Hameister et al., 2020). The implant itself will behave differently in the presence of the disease-induced microenvironment changes, namely by increased metal ion leaching (Arteaga et al., 2021). Hyperlipidemia, frequently present in diabetic patients and in several other diseases, as well as a primary condition, causes overproduction of ROS *in vitro*, interfering with the Wnt/β-Catenin pathway and causing osteoblast dysfunction; *in vivo*, poor bone formation at the bone-implant interface was observed (Wang Y. N. et al., 2021). Overproduction of ROS may also cause eNOS uncoupling, and a shift from NO production to O_2_^−^, contributing to oxidative distress.

Strategies to curb the deleterious effects of the excess ROS may include implant surface modification by coatings with antioxidant properties or by nanotopographic-modulated FoxO activation (Abuid et al., 2021; Huang et al., 2021; Shao et al., 2021); protein adsorption to the implant surface, and subsequent changes in the protein structure influence cell adhesion, metal particles release and ultimately, implant stability (Rahimi et al., 2021).

Biomaterials’ selection should considerer their contribution to optimum redox state, as suggested by Sthijns et al. (Sthijns et al., 2021), who evaluated different polymers for a pancreatic islet encapsulation device; cell sensitivity to oxidative stress is variable and essential for cell survival, proliferation, and function (Sthijns et al., 2021), and contributes to avoid chronic inflammation and peri-implantar fibrosis. As reviewed by Sthijns et al. (Sthijns et al., 2018), in tissue engineering approaches, ensuring adequate perfusion of the construct is paramount for cell survival and to avoid increased ROS formation. Several strategies may be pursued to achieve optimum delivery of oxygen while avoiding its toxicity. Developing biomaterials that are able to modulate the redox-balance systems such NADPH oxidase, the Nrf2 or the HIF pathways, is a promising approach (Sthijns et al., 2018).

On the other hand, there is also an increased interest in how the redox state may be used as a stimulus in the development of active implants, tuning their response to the local microenvironment. Redox-responsive biomaterials are being studied for drug delivery, with promising results (Yang et al., 2014; Cheewatanakornkool et al., 2018; Gong et al., 2018; Yu et al., 2020). A bio-inspired approach to infection control through active implant generation of ROS has been tested. In this case, implants were responsive to local pH decreases (Song et al., 2020).

Promoting bone health should also encompass nutrition. The most abundant dietary antioxidants are polyphenols and anthocyanin, present in fruits, vegetables, cereals, dry legumes, chocolate, tea, coffee, and wine. As reviewed by Domazetovic et al. (Domazetovic et al., 2017), antioxidant intake promotes bone health. In animals and selected groups of patients with osteoporosis or inflammatory bone diseases, the use of antioxidants was beneficial for the treatment and prevention of bone loss (Domazetovic et al., 2017). Total dietary antioxidant capacity has been described as inversely associated with the risk of osteoporosis in postmenopausal women and positively associated with bone mass in both pre- and postmenopausal women (Kim and Park, 2021). Eicosapentaenoic acid and docosahexaenoic acid omega-3 fatty acids reduce superoxide production catalyzed by the NADPH oxidase in neutrophils, and there is growing evidence of the benefits of dietary supplementation in lowering the expression of catabolic and inflammatory genes in osteoarthritis. Omega-3 fatty acids reduce oxidative stress and apoptosis *via* the NF-κB and the iNOS pathways. Dietary fatty acids may modulate osteogenic differentiation in mesenchymal stem cells by up-regulation in phosphorylation of protein kinase B (Akt) at the plasma membrane (Wauquier et al., 2009; Abshirini et al., 2021).

Literature concerning the role of oxidative stress in mechanotransduction and adaptive responses in endothelial cells is more abundant and allows a better understanding of both the mechanical forces involved and the cellular response (Hsieh et al., 2014; Chatterjee, 2018; Roux et al., 2020; Psefteli et al., 2021). Some studies in cartilage also contribute to the characterization of mechanical loading and redox state alterations (Coleman et al., 2017; Walsh et al., 2019), with evidence supporting that inflammation induced by mechanical loading is ROS-mediated (Kamalathevan et al., 2021).

There is, however, surprisingly limited knowledge on the influence of the biomechanical environment in the bone and bone-implant interface on the redox balance, with some of the information available based on maxillofacial implantology focusing on the effects of the implant itself. It is recognized that ROS boost the inflammatory response and cause both RANKL-induced osteoclastogenesis and osteoblast apoptosis, leading to periprosthetic osteolysis aseptic loosening, the most frequent cause of implant failure (Borys et al., 2018; Ozawa et al., 2020; Galliera et al., 2021). Bone unloading such as observed in disuse and microgravity conditions leads to decreased bone mass and quality, through redox-mediated pathways. Implants, especially metallic ones, have mismatched elastic modulus when compared to the bone, leading to altered loading patterns (stress shielding and stress concentration). Therefore, it would be desirable to understand the contribution of such altered strain levels to oxidative stress reported in numerous studies.

A recent systematic review by Kohli et al. summarizes the considerable overlap between the values of micromotion associated with osseointegrated implants vs failed ones (mean value of micromotion of 112 ± 176 μm for implants showing osteointegration versus 349 ± 231 μm for non-integrated). Other works correlate the implant stability with fibrous tissue formation at the interface, associated with micromotion and lower implant stability (Ramos et al., 2013). The need to consider the loading conditions in detail, combined with the different localized bone-implant geometry, as well as the fundamental mechanobiology mechanisms was also highlighted (Kohli et al., 2021).

The ultimate strength of bone has been reported 25,000 μ, with physiologic bone loading ranging from 200 to 2,500 µ. Peak strains above 2,500 lead to increased bone mass. However, repeatedly loading bone above 4,000 µ leads to accumulation of damage. Repeated loading within the physiological bone promotes adequate bone remodeling and bone mass maintenance (Roberts et al., 2004; Ramos et al., 2015).

Mechanical unloading downregulates bone mass *via* intracellular ROS generation (Morikawa et al., 2013). There is evidence that appropriate mechanical stimulation improves antioxidant functions in mesenchymal stem cells and improves bone regeneration, while excessive stretch is deleterious for the cellular antioxidant mechanism. The effect of cyclic stretch at magnitudes of 2.5, 5, and 10% of human bone marrow-derived mesenchymal stem cells was evaluated in terms of proliferation, ROs, antioxidant enzymes expression, and osteogenesis. Osteogenesis was increased by 5% stretch, intracellular ROS decreased and the levels of SOD1 and SOD2 increased with 2,5 and 5% stretch; with the same levels of stretch, ROS scavenging enzymes CAT and GPx1 were also increased. The stretch-induced antioxidant effect was through activation of the adenosine monophosphate-activated protein kinase-silent information regulator type 1 signaling pathway, was more pronounced with 5% stretch and null under 10% stretch (Chen et al., 2018). It would be useful to understand if PGC-1α is involved in the increased SOD expression in response to different strain levels in bone cells, as it happens in muscle.

Acknowledging the consequences of the disruption of the loading patterns to bone health, and in face of what is already known, the link between mechanical stimulation and local redox state is apparent. However, although the cellular pathways involving ROS signaling are more and more characterized, the bond of the mechanical environment in relation to the redox state is yet to be fully explored. Computational models may help predict not only ROS production but also ROS scavenging and the influence of inhibitors on ROS production (Gauthier et al., 2013; Pereira et al., 2016). However, although this is a promising approach and several of the existing models were later confirmed experimentally, the incorporation of the modulation of multiple signal-transduction cascades into a dynamic model is still lacking (Pereira et al., 2016). An ideal model should be able to consider spatial effects too, since ROS may originate in different parts of the cell, in addition to predicting ROS damage and signal modulation (Guimera et al., 2019). Haack et al. (Haack et al., 2015) developed a stochastic computational model of canonical WNT/β-catenin signaling, combining membrane-related and intracellular processes, including lipid rafts/receptor dynamics as well as Wnt and ROS dependent β-catenin activation, to investigate its influence in the early phases of neural differentiation (Haack et al., 2015). Signaling through this same pathway increases bone mass by the renewal of stem cells, stimulation of preosteoblast replication, osteoblastogenesis, and inhibition of osteoblast and osteocyte apoptosis. So, along with the application of the existing models to bone cells, further developing models integrating mechanotransduction and redox state is a much-needed, logical approach.

In what measure strain, stress, frequency of stimuli, and rest periods are translated into redox state changes, intermediated by the different reactive species, is a major challenge that may be addressed through in-silico approaches, as well as experimentally. However, as every unknown, it also carries a promise of therapeutic applications through multimodal approach, including optimal physical stimuli, adequate antioxidant nutritional support, device selection and modification to minimize oxidative distress and eventually, drugs specifically targeting redox state and redox-sensitive pathways.
